# Supplementary material for: Health care providers’ perceptions and experiences related to Midwife-led continuity of care–A qualitative study
Source: PLoS One. 2021 Oct 14;16(10):e0258248. doi: 10.1371/journal.pone.0258248 (PMC8516211; doi:10.1371/journal.pone.0258248)
Supplement: S1 Text — (DOCX) [file pone.0258248.s001.docx]

**S1 Text: Focus group discussion and in-Depth Interview guide for health care providers**

**UNIVERSITY OF GONDAR**

**COLLEGE OF HEALTH SCIENCES**

**SCHOOL OF MIDWIFERY**

Information sheet for FGD and In-depth interview participants for the study entitled: **Health care providers’ perceptions and experiences related to Midwife-led continuity of care in north Shoa zone, Amhara region, Ethiopia**: **a qualitative study**

**Participants:** Midwives for FGD and other health care professionals (obstetricians, Emergency surgical officers and medical doctors) who are working in maternal health care unit for in-depth interview.

**Investigators:** Mr. Solomon Hailemeskel, Professor. Kyllike Christensson, Dr. Helena Lindgren, Dr. Kassahun Alemu and Dr. Esubalew Tesfahun

**Part I – Information sheet**

***Introduction***

Good morning/afternoon! My name is _________________. I represent the research team from University of Gondar. We are speaking with pregnant women about the follow up study on model of maternal health care they received on the current pregnancy and the contribution of these models and care on maternal and neonatal outcomes. We are also trying to study the health care providers’ perceptions and experiences related to Midwife-led continuity of care.

***Purpose of the research***

A number of problems can occur and endanger the life of mothers during pregnancy and child birth. To deal with these problems, assessing the effectiveness of the model of maternal health care in which pregnant mothers received has been an effective intervention. Currently, there are different model of maternal health cares practiced in different countries, of these models midwife led maternal care is one of the commonly practiced one in developed countries. However, in our country its status and contribution on maternal and newborn health haven‘t been well assessed. Therefore, this study is aimed to fill this gap by conducting follow up study on pregnant mothers in North Shoa Zone.

***Procedures***

You are selected because you are working in maternal and chilled health unit. I will have some discussion questions about the model of maternal health care, continuum of care and its challenges and strengths. We will have also discussion about your experience and perception on midwife-led continuum of care. So, I request your volunteer participation.

***Risks and discomfort***

There might be slight discomfort to share some personal information. However, we do not wish this to happen and you may refuse to answer any of the questions if you feel uncomfortable.

***Benefits***

There will be no direct benefit to you, but based on the information you provide us, we will design strategies to improve the interventions targeted to develop the most appropriate model of maternal health care for Ethiopian women so as to prevent maternal and neonatal health problems occurring during pregnancy and child birth.

***Confidentiality***

The information that we collect in this study will be kept confidential. Your name will not be written in this form and the information we collect from you will not be shown to anyone. The collected data will also be used in aggregated form. The hard copies will also be kept in a locked cabinet and will not be divulged to anyone, except the investigators.

***Right to refuse or withdraw***

You can refuse to answer any question to which you are not comfortable. You may stop participating in the discussion at any time if not convenient for you without losing any of your rights as a participant. However, your active participation in the discussion and genuine responses for the interview has paramount importance in improving maternal health services in future.

**Certificate of consent**

With due understanding of the aforementioned information, are you willing to participate in the study?

Yes

I have been requested to take part in the research and the foregoing information has been read to me. I have had the opportunity to ask questions about it and any questions I have been asked have been answered to my satisfaction. I consent voluntarily to participate in this study and understand that I have the right to withdraw from the interview and follow up at any time without in anyway affecting my right.

**Signature of the participants**

1. Signature________________ date _______________

2. Signature________________ date _______________

3. Signature ________________ date _______________

4. Signature ________________ date _______________

5. Signature ________________ date _______________

**(Proceed with the interview)**

No **(Terminate the interview)**

**Signature of the Moderator: Name ______________, Signature_________ date**

**Signature of the Note taker: Name _______________, Signature_________ date**

**Supervisors/Researcher remark and signature**

Name ________________ Signature ________________ date _______________

Note: In case of any unclearity you can communicate the principal investigator through the telephone Number: +251-0913-31-29-12.

I-Participants back ground

(Don‘t write their name, code as P1, p2, p3….p12 under the column headed discussant code)

| S/N | Discussant code | Age | Educational background/ profession/ | Work experience | Working unit |  |
| --- | --- | --- | --- | --- | --- | --- |
|  |  |  |  |  |  |  |
|  |  |  |  |  |  |  |
|  |  |  |  |  |  |  |

**Objective of the study:** to explore health care provider’s perception and experience on midwife-led continuity of care model.

Date: _______________ Time:________________ Place:________________

Facilitator: __________________ Note taker: _________________ Recorder:______________

**Focus group guide and In-depth interview questions**

1. How do you understand model of maternal health care? What does it mean for you?
2. What type of model of care you practice in your hospital?

**Probe: How did the midwifery care practice organized in your health facility?**

1. What are the challenges of the current model of maternal health care practiced in your Hospital?

**Probe: would you please elaborate your concept in relation to quality of maternal care, in relation to provider’s professional autonomy**

1. How do you see the effect of the model of care you practiced in your facilities for the health of the mother?

**Probe: Do you think that the current model of maternal health care contributes to improve maternal health care to its expected level?**

1. How do you explain your contribution as a midwife for the improvement of maternal health care?
2. What do you think should be done to contribute more for maternal health care (related with model of care?)
3. Do you think that implementing midwife-led continuum of care helps to improve problems of maternal health care?

**Probe: How do you explain the concept of continuity of maternal health care?**

**Probe: what are the characteristics of midwife continuum of care?**

**Probe: does your model of care practiced in your health facility fulfilled the three characteristics of continuum of care model (continuity of information, continuity of care provider and continuity of care management?)**

1. Is that possible to implement midwife-led continuum of care in our country? How?

**Probe: if we implement midwife-led continuity of care model what will be the possible challenges we might face?**

1. Does having continuity of midwifery care model has contribution for the improvement of maternal health care?

**Probe: if yes how it improves maternal health care?**

**Probe: What makes it different with other model of care?**

1. Are you as a midwife ready to work with midwife-led continuity of care model?

**Probe: what makes you interested?**

1. Anything you want to add, please welcome

**I have finished my questions thank you very much for your time and response.**
