# Supplementary material for: Health care providers’ perceptions and experiences related to Midwife-led continuity of care–A qualitative study
Source: PLoS One. 2021 Oct 14;16(10):e0258248. doi: 10.1371/journal.pone.0258248 (PMC8516211; doi:10.1371/journal.pone.0258248)
Supplement: S2 Text — (DOCX) [file pone.0258248.s002.docx]

**S2 Text: Focus group discussion and in-depth interview participant’s characteristics**

| **Number** | **Age** | **Sex** | **Level of education** | **Working unit** | **Work experience** |
| --- | --- | --- | --- | --- | --- |
|  | 29 | M | BSc midwife | Delivery | 5 years |
|  | 29 | M | BSc midwife | Delivery | 5 years |
|  | 24 | F | BSc midwife | Delivery | 1 year |
|  | 26 | M | Diploma midwife | Delivery | 3 years |
|  | 27 | M | BSc midwife | Delivery | 4 years |
|  | 29 | M | BSc midwife | ANC | 6 years |
|  | 28 | M | BSc midwife | Gyn/obs | 4 years |
|  | 27 | M | BSc midwife | Gyn/obs | 3 years |
|  | 27 | M | BSc midwife | Gyn/obs | 5 years |
|  | 25 | M | BSc midwife | Gyn/obs | 5 years |
|  | 25 | M | Diploma | Gyn/obs | 2 years |
|  | 23 | F | BSc midwife | Gyn/obs | 1 years |
|  | 28 | M | BSc in midwifery | Labor and delivery | 3 years |
|  | 33 | F | Diploma | ANC | 10 years |
|  | 26 | M | BSc in midwifery | ANC | 2 years |
|  | 25 | M | Diploma in midwifery | Labour and delivery | 1 year |
|  | 27 | F | BSc in midwifery | Labour and delivery | 5 years |
|  | 26 | F | MSc in midwifery | ANC | 3 years |
|  | 24 | M | BSc in midwifery | Delivery | 3 years |
|  | 25 | M | BSc in midwifery | Delivery | 2 years |
|  | 25 | F | BSc in midwifery | Postnatal | 3 years |
|  | 25 | M | BSc in midwifery | ANC | 2 years |
|  | 25 | M | BSc in midwifery | Delivery | 4 years |
|  | 26 | M | BSc in midwifery | Delivery | 3 years |
|  | 26 | F | BSc in midwifery | Family planning | 3 years |
|  | 32 | M | IESO | All MCH unit | 9 years |
|  | 35 | M | IESO | All MCH unit | 9 years |
|  | 25 | M | MD | All MCH unit | 3 years |
|  | 24 | M | MD | All MCH unit | 4 years |
|  | 23 | M | MD | All MCH unit | 4 years |
|  | 25 | M | MD | All MCH unit | 4 years |
|  | 30 | M | IESO | All MCH unit | 7 years |
|  | 29 | M | IESO | All MCH unit | 8 years |
