# Supplementary material for: Health care providers’ perceptions and experiences related to Midwife-led continuity of care–A qualitative study
Source: PLoS One. 2021 Oct 14;16(10):e0258248. doi: 10.1371/journal.pone.0258248 (PMC8516211; doi:10.1371/journal.pone.0258248)
Supplement: Attachment — Submitted filename: Response to Reviewers.docx [file pone.0258248.s003.docx]

**Answer to the editor's comments:**

A rebuttal letter, with point-by-point responses to yours and the reviewers' comments is also provided. The following order has been applied for the submission: Response to Reviewers; Revised Manuscript with Track Changes; Manuscript. Besides and interview guide, a professional language editor has gone through the manuscript and checked for grammar, spelling and typological errors except flow and understanding of the content.

1. Please ensure that your manuscript meets PLOS ONE's style requirements, including those for file naming.

Answer: Thanks and we made revision on the revised manuscript based on PLOSE ONE style

2. Please provide the following information in your Methods section relating to your qualitative methodology:

i) The expertise and training of the interviewers and

Answer: The interview was done by one of the principal investigator and two data collectors. The data collectors had MSc in maternal and reproductive health. During the interview we used recorder and note taker. The interviewers had practiced the question on how to interview during the time of piloting the interview tool.

ii) How the focus groups and discussion groups audio recordings were translated for analysis.

Answer: After we hear the audiotaped record repeatedly translation was made by the principal investigator together with the data collectors who conducted the interview verbatim. We had also made triangulation with the notes we had during interview. The translated data were also cross-checked with the audio file to ensure its proper transcription and translation.

Finally, please provide the interview guide used as a part of the study as Supporting Information.

Answer: thanks and we had attached the interview guide we used as a separate file in the revised manuscript.

2. Please remove your figure from within your manuscript file, leaving only the individual TIFF/EPS image file, uploaded separately.  This will be automatically included in the reviewers’ PDF.

Answer: it is removed in the revised manuscript and attached as a separate file

3. Your ethics statement should only appear in the Methods section of your manuscript. If your ethics statement is written in any section besides the Methods, please delete it from any other section.

Answer: thanks and we removed it for the revised manuscript

**Letter to reviewers**

**Review Comments followed by answers**

Reviewer #1: An interesting paper and important area of research. The manuscript is sound however it needs attention to the details of the study and justification of themes. Also need to be sure to include study details around questions and clarity in how these were developed and validated prior to the study.

Answer: Thanks for reading and providing us with helpful insights. All your valuable comments have been taken seriously and made correction on the manuscript accordingly. The themes are identified based on the participant’s response and the research question we need to answer. The interview questions were developed based on literature review and Ethiopian health care organization context. And the tool was piloted by the same study participants prior to actual data collection.
Referencing needs to be consistent through the manuscript and quote original sources eg ref 1 quotes WHO guidelines for ANC to justify midwives as primary care providers – which is true but not from the guidelines (they include the primary reference in the guidelines). Need to reference all assertions.

Answer: thank you so much for your valuable insights and correction is made on the manuscript by referencing the original source.

Also consider the scope of your work as exploratory and provides further unknown information to inform delivery of maternity services (too much detail is provided in this paper about what that could be).

Answer: thanks it seems too much detail but what we have done was on exploring the health care providers view and practice on model of maternal health care. On this regard we had asked them about the existed model of care practiced in their health facility and we had also asked them about the new model of maternal health care (midwife-led continuity of care) what they feel about it and their understanding and willingness to work with this model of care. After we get their response we did a quasi-experimental study using the MLCC to evaluate its effect on maternal and neonatal outcomes.

I have reviewed and noted the following points you may wish to consider to further improve your manuscript.

Introduction
would be best to cite original research not guidelines ie midwives primary care providers in many countries – true so find original sources, and several to make this point. See earlier point

Answer: thanks for this and we made correction and the original research is referenced in the revised manuscript

Explain what mid led care is – so it is clear to the reader.

Answer: In MLCC models, a known and trusted midwife (caseload midwifery), or small group of known midwives (team midwifery), supports a woman throughout the antenatal, intrapartum and postnatal period, to facilitate a healthy pregnancy and childbirth, and healthy parenting practices

Need references for all your assertions eg line 71 -74 need references for each of these you state

Answer: thanks for this and we made correction and reference is inserted accordingly on the revised manuscript

Line 80. Add what happens if antenatal deviates from usual physiological parameters (and reference)

Answer: thanks and we add the following explanations in the revised manuscript with its appropriate reference. “The model also includes identifying, referring and coordinating care for women who require obstetric or other specialist attention. Thus, the MLCC model exists within a multidisciplinary network in which consultation and referral to other care providers occurs when necessary”

Line 145 explain when you stop and how know enough for your sample (and reference). It is included but as last sentence, meaning justification and clarity could be improved with rewriting.

Answer: we end up the FGD and interview when we feel that there is point of saturations. The point of saturation was confirmed when there is no new idea generated form the study participants.

151 what were the themes of the questions; include example of the questions you asked. How did you determine these questions and test prior to the interviews? These will help explain and describe your findings later – the link is not clear

Answer: thanks for this and the interview guide we used during data collection is provided as a supplementary file in the revised manuscript. Regarding the process of developing the interview questions, the question were determined with detail discussion with research team and it was pretested before the actual data collection. And all these process has been stated in the manuscript.

163 do you mean written informed consent?

Answer: we explained the objective of the study and anonymity of their response and we ask orally their agreement to participate in the discussion and interview. After we obtained their oral consent we undertake the interview.

166 Data analysis process include but make briefer and no need for bolding of steps

Answer: thanks bolding of the steps are corrected in the revised manuscript. Regarding analysis method we used thematic analysis as described by Braun and Clarke. And we tried to explain the five most important steps of thematic analysis.

186 include the approval number in the manuscript

Answer: The study was approved by the Institutional Review Board at the University of Gondar with this reference number (O/V/P/RCS/05/1050/2019 on March 2019). And of course it was included in the last page of the manuscript at line number 489 under the sub heading Ethics approval and consent to participate.

198 explain what a primary hospital is – size, services – I may have missed this earlier?
Answer: the primary hospitals were explained under the study area. To explain more the primary hospitals are located at woreda level (the lowest administrative unit in Ethiopia) and expected to provide service for 100,000 populations with a capacity of 100 beds. The hospitals will provide all health services.

201 Overarching theme is very broad (and includes sub section re workload).
Themes need justification as to their identification. Then having identify two themes – are these theme A & B. Be consistent with how describe ie keep as theme one (?A) and theme two (?B). You are very aware of the work it is new to readers so be sure to step through this clearly and methodically.
Answer: thank you for this and it is revised and labeled in the revised manuscript. As you said we have identified one extended them and two themes and five sub themes

214 label the diagram as per your themes

Answer: the diagram is labeled as per the themes in the revised manuscript

247 need to edit and number these so themes and subthemes are very clear

Answer: the revised manuscript is edited and number is given for each themes and subthemes.

285 + Code2 – is midwifery? Obstetric? Consider identifying the occupation for each

Answer: thanks it was coted from midwifery participants and all participants’ occupation is included in the revised manuscript

Reference list – include doi or online access information

Answer: we used endnote software for reference and we updated the reference using the software to include all important lists in the reference in the revised manuscript. We add DOI for the references in the revised manuscript.

Reviewer #2: I would like to thank the authors for the opportunity to review this manuscript. This is a very important topic with clear implications for practice, and I believe this work will be a valuable contribution to the literature. There are however, several issues with the manuscript itself that I believe need to be addressed before I can recommend it for publication.

Answer: thank you so much for your time and your valuable comments provided for the manuscript. It will help us to improve the quality of the manuscript. And we really appreciate your contribution.

General comments:

1. It is my opinion that the overall length of the manuscript (across all sections) could be reduced. Many sentences throughout could be abridged/shortened while retaining the same level of detail. Please consider this is your resubmission.

Answer: According to PLOSE ONE journal guideline manuscripts can be any length and there are no restrictions on word count. But we strongly believe to have a short and concise manuscript. For this reason we gave the final manuscript for English language editor and we made some corrections to shorter the word counts of the final manuscript.

Introduction:
2. Page 4, row 74: please just use the acronym “MLCC” here as full name is already spelled out in row 71.

Answer: Thanks for this and it is corrected in the revised manuscript

3. Page 4, row 81: please clarify what you mean by “the standard model of care”.

Answer: standard model of care is the already existed model of care in our country and characterized by care is provided by different health care providers. In this model of care the mother will receive the care by different health care providers from antenatal care up to postnatal care. And it is explained in the revised manuscript.

4. The authors discuss the ideal of making MLCC possible for all women. Therefore, I think a statement of how pregnancies deemed higher-risk (thus requiring the care of an obstetrician) would fit in to this model, is important.

Answer: there are studies reported that midwife-led continuity of care are helpful for high risk mothers. Besides the model allows two to three obstetrician visit if the mother wants to have and if there is complication that needs higher level of care. But in our study we want to focus on low risk mothers only because it is a new model of care for our country we just want to start with simple and routine care which can be provided by middle level health care providers.

5. As definitions of MLCC often differ, can the author’s please clarify their views on the number of midwives that would operate together/that each woman gets to know, in the model they are proposing? This is particularly important when considering what would happen if a woman’s known midwife was not available when needed– i.e., does the woman actually get to know a small group of midwives in the event her main midwife is not available for birth? If the author’s believe that these details should be determined at the point of MLCC implementation, please provide a statement clarifying this and/or discuss the different options that exist.

Answer: yes there are different arrangement MLCC. Of these the commonest are caseload midwifery model in which a single midwife will have a limited number of mother to follow and provide care. In this model there is a backup midwife who will replace the caseload midwife in her/his absence. The other type of model is group midwifery care where a small group of midwives usually 4 to 8 midwives work together and provide care for pregnant mothers. In this case all of the midwives working together should be known by the mother before the beginning of the care. They plan together and they work out in harmony. In our case we choose group midwifery continuity of care. We organize the midwife in a group of four and they plan together and provide care.

Methods:
6. Page 7, row 150: It is unclear if saturation was used to determine the sample size or if the authors just happened to reach saturation with the sample they were able to obtain. Please reword this sentence to clarify.

Answer: actually there is no hard and fast rules to determine the sample size for qualitative studies. We used saturation of information and redundancy (until new categories, themes or explanations stop emerging from the data) to determine the sample size and we explained this in the revised manuscript.

7. Page 7, row 156: please clarify what ‘Group-2’ is.

Answer: group two is health care providers who are working in maternal health care unit who are not midwives. These groups include integrated emergency surgical officers, medical doctors and others. And we revise it in the revised manuscript

8. Data Collection: A brief description of the content of the interview and discussion guides is needed. It is difficult to interpret findings without knowing the types of questions that were asked/discussion points raised. e.g., did you provide the participants with a definition of MLCC? The full interview and discussion guides should also be provided as Appendices/Supplementary material.

Answer: thanks for this and the interview guide we used during data collection is provided as a supplementary file in the revised manuscript. Regarding the process of developing the interview questions, the question were determined with detail discussion with research team and it was pretested before the actual data collection. And all these process has been stated in the manuscript.

9. Data Analysis: Please specify how many people were involved in each phase of the thematic analysis and whether duplicate coding was done etc.

Answer: four of the investigators were involved in all stages of the data analysis. The collected data was transcribed by the investigators. Code and meaning units were identified by three of the investigators and we set together and decide on the identified meaning units. Then we grouped the identified meaning units together based on its content and we identify themes and extended themes. In all these process all the investigators were involved and decide together.

Results:
10. I am worried that the results in current format may jeopardies participant confidentiality. For instance, the author’s note that all eligible emergency surgical officer’s (4/4) participated, and then each of the four participating hospitals are named in the results. For reader’s familiar with these hospitals, would it be obvious who these ESO’s were? There are also very low numbers (<5) of some participant groups outlined in Table 1. Please reconsider/revise some of the data presented in light of these potential confidentiality implications.

Answer: Thank you so much for your concern and really we as an investigator tried all our best to maintain the confidentiality of the respondents. For this reason the data was collected in four primary hospitals. In these hospitals there are eight (two in each hospital) emergency surgical officers. Of these we interviewed one in each hospital. Hence, it will not beak the participant’s confidentiality.

11. The authors refer often to ‘participants’ when discussing focus group/interview results. Here, it would be helpful to know specifically, if they are referring to midwives, doctors, or ESOs (or a combination of these). A strength of this work is that different types of providers were included. Ideally, this should enable the author’s to determine the concordance/discordance of views between these different provider groups.

Answer: for this we added the provider’s occupation in the coted text to refer whose idea is it.

12. The wording of the overarching theme ‘Midwives welcoming continuity of care despite concerns about organization and workload’ comes across as being purely based on the midwives’ views and not inclusive of those in the other provider groups interviewed. I assume the overarching theme is based on the entirety of data analyzed, so please consider rewording this theme or explain if I have misunderstood something.

Answer: thanks for your insight. You are correct it seems the theme only reflects the midwives view. But what we think was in MLCC model the main care provider are midwives. They receive the mother, plan together and provide all maternal health care autonomously. We believe the theme should reflect the view of these professionals. But in the course of care provision they may communicate with other health care providers to get their help because maternal health care needs team work. Hence to start implementation of MLCC model the view and willingness of midwives to work with this model of are is very important than other health care providers. Of course to be successful other health care providers are also important for the model.

Discussion:
13. Page 18, row 375: There appears to be some repetition in this paragraph. Please review and revise.

Answer: thanks it is revised and some idea which seems repetition is corrected in the revised manuscript.

14. Page 19, row 397: some errors in this sentence, please review and revise.

Answer: thanks it is corrected in the revised manuscript.

15. Page 21, row 449: “no generalizations to larger populations can be made” - please explain this further. Why do you think your sample was not representative of the general population of midwives, doctors, and ESOs in Ethiopia?

Answer: yes it would be representative for the study area but it may not be representative for the larger population. This is because the number of study participants may not be enough to represent the larger population in terms of number, year of experience, level of qualification, working are (our is only primary hospital, but there are also health centers and referral hospitals in the country). Besides, People differ in their experience and understandings of the reality and a social phenomenon cannot be understood outside its own context hence generalization is not the goal of qualitative study.

16. It would be interesting to know the author's views on the effectiveness of the focus groups vs. interviews? Do you think they yielded the same quality of data? Based on this, do you have recommendations for optimal methodology of future work?

Answer: both techniques of qualitative data collection are effective. What matters is the objective of the study and the research question we want to answer. For example if we want to answer the view and perceptions of people FGD would be helpful on obtaining reach expression of them. In our case for example, is what are the commonly held views of midwives who are working in maternal health care unit about MLCC? In this case we used FGD as a tool. Whereas we used individual interview with other non-midwife professional because they are small to conduct FGD. So what we recommend is to use FGD or interview when you feel that is appropriate to answer your research questions.

17. More specific recommendations for next steps (e.g., what questions still need to be answered? What type of studies should be conducted?) Would strengthen the Discussion.

Answer: thanks for this and we recommend implementation research to be done in the future to investigate the effectiveness of the model in Ethiopian context. We tried to explore the view and experience of health care providers who are working on maternal health care unit on the model of maternal health care. Hence, the current research did not answer what will happen on the maternal and health care provider’s side when implementation of the new model of care is started. Because working in actual setup and expressing the view and experience are quite different.

Thank you so much once again for your valuable comments and time you spent to evaluate my research work.

With kind regards,

Solomon Hailemeskel (PI)
