# Supplementary material for: Health care providers’ perceptions and experiences related to Midwife-led continuity of care–A qualitative study
Source: PLoS One. 2021 Oct 14;16(10):e0258248. doi: 10.1371/journal.pone.0258248 (PMC8516211; doi:10.1371/journal.pone.0258248)
Supplement: Attachment — Submitted filename: Response to Reviewers.docx [file pone.0258248.s004.docx]

**Answer to the editor's comments:**

A rebuttal letter, with point-by-point responses to yours and the reviewers' comments is provided in this revised version of the manuscript. The following order has been applied for the submission: Response to Reviewers; Revised Manuscript with Track Changes and Manuscript without track change.

Journal Requirements:

Please review your reference list to ensure that it is complete and correct. If you have cited papers that have been retracted, please include the rationale for doing so in the manuscript text, or remove these references and replace them with relevant current references. Any changes to the reference list should be mentioned in the rebuttal letter that accompanies your revised manuscript. If you need to cite a retracted article, indicate the article’s retracted status in the References list and also include a citation and full reference for the retraction notice.

Answer: yes we revised the reference list based on the journal reference style and we used endnote reference software. We have also tried to replace some of the old references with the recent and most appropriate one. We have checked all the references for retracted paper and all reference lists we used were not retracted. Based on our recent search we have replaced the following reference with the recent reference.

1. Hatem M, Sandall J, Devane D, Soltani H, Gates S. Midwife‐led versus other models of care for childbearing women. Cochrane database of systematic reviews. 2008;4. doi: 10.1002/14651858.CD004667. is replaced by Sandall J, Soltani H, Gates S, Shennan A, Devane D. Midwife-led continuity models versus other models of care for childbearing women. The Cochrane database of systematic reviews. 2013;(8):Cd004667. Epub 2013/08/22. doi: 10.1002/14651858.CD004667.pub3. PubMed PMID: 23963739.
2. World Health Organization, UNICEF. Reduction of maternal mortality: a joint WHO/UNFPA/UNICEF/World Bank Statement. 1999. Is replaced by Muna A, Mohamed A , Zalha A, Sarah BZ, Luc B, Mathieu B, et al. the state of world midwifery report. UNFPA, ICM, WHO, 2021.
3. Reference number 13: PMNCH, WHO, and WB, AHPSR. Success factors for women's and children's health: policy and program highlights from 10 fast-track countries. Geneva: WHO. 2014. Is remuved and Williams K, Lago L, Lainchbury A, Eagar KJM. Mothers’ views of caseload midwifery and the value of continuity of care at an Australian regional hospital. 2010;26(6):615-21. Is added to the reference list
4. Reference number 45: McCourt C, Page L, Hewison J, A. V. Evaluation of one-to- one midwifery : women responses to care. Birth. 1998 Jun;25(2):73-80. doi: https://doi.org/10.1046/j.1523-536x.1998.00073. Is remuved from the reference list because the idea explained by this article can be adressed by other references used in the manuscript.

Additional Editor Comments (if provided):

As you will see I have received comprehensive comments from one of the Reviewers. I think these suggestion are comprehensive are substantial and would ask that you address them fully. Bear in mind comments re Q9.

Answer: Thanks and we made revision on the revised manuscript using track changes based the suggestions provided by the reviewers and also we prepared point by point responses for further elaboration on the questions in the following pages.

**Letter to reviewers**

**Review Comments followed by answers**

Reviewer #1: The manuscript is coming along and reads better this version. A few minor amendments are suggested to improve further for publication standard:

Answer: Thanks for reading and providing us with helpful insights. All your valuable comments have been taken seriously and made correction on the revised manuscript accordingly.

Line 91 & 92 needs reference

Answer: thank you so much for your valuable insights and correction is made on the manuscript by referencing the original source. These are the references we used in the revised manuscript.

1. Sandall J, Soltani H, Gates S, Shennan A, Devane D. Midwife‐led continuity models versus other models of care for childbearing women. Cochrane database of systematic reviews. 2016;(4). doi: https://doi.org/10.1002/14651858.CD004667.pub5.
2. Zeitlin J, Mohangoo AD, Delnord M, Cuttini M. The second European Perinatal Health Report: documenting changes over 6 years in the health of mothers and babies in Europe. J Epidemiol Community Health. 2013;67(12):983-5. Epub 2013/09/21. doi: 10.1136/jech-2013-203291. PubMed PMID: 24052513.

Line 98 when did “traditional” obs/hosp care start

Answer: In Ethiopian context the only practiced model of care is known as shared model of care (where every health care provider assigned in maternity unit will provide the care for the mother). And this kind of model of care was practiced since the establishment of modern medicine in Ethiopia.

Line 147 – for those who birth at home is it midwifery led?

Answer: No, for pregnant mother who delivered at home; the traditional birth attendants or relatives will attend her birth. In Ethiopian context Midwives are assigned only at health facilities starting from Health center to referral hospitals. Hence, if the pregnant mother gave birth at home there will not be the chance to be attended by the midwives and other health care providers. This is why we tried to investigate the effectiveness of midwife-led continuity of care model to reduce the rate of home birth. As we have clearly put the percentage of institutional delivery in the study area only 53% of pregnant mother gave birth at the health facility, the remaining 47% of them gave birth at home nonskilled birth attendants.

Line 163 Details about research team: a) what are interviewers experience and training in interviewing for data collection?

Answer: the principal investigator used to collect the data with two midwifery professionals with MSc in maternal and reproductive health background (one for note taker and the other recorder). But, the principal investigator facilitated the interview with the participants. For this purpose the data collector trained about the interview questions and they practiced it from other similar participants before the actual data collections. Hence, we made correction on the revised manuscript and it is stated in the data collection section of the manuscript about the data collector’s expertise on data collection and that training was given to them before the data collection on the revised version of the manuscript.

b) Relationship with the participants? Are any known to researcher? What relationships existed or were established around this work.

Answer: thanks for this and we made correction on the revised manuscript as both the principal investigator and the data collectors do not have any relationship with the study participants. All the data collectors are not know to the participants and have no any previous relationship that would affect the data collection process. And of course the data collectors assured the anonymity of the data for the participants before they began the interview.

Line 172 – overview broad question asked to the group to context for the reader

Answer: thanks for this and we have attached as a supplementary file the interview and FGD guide we used in this study. We used the following questions as an introductory question to provoke discussion: “would you please explain about the type of model of maternal health care practiced in your hospitals and How do you understand the concept of model of maternal health care?” and we continue our interview based on the participants response by using probing questions until we gate reach data on the research question we want to answer.

Line 185 – Were participants given opportunity to provide feedback/ amend/ approve their transcripts prior data analysis?

Answer: yes since the principal investigator undertake the interview there was a chance to transcribe the audio tape recode before we collect the other FGD and interview.

Line 186 how many & who involved in coding? – I note this was raised by another reviewer also. Your response is provided in the response to reviewers but has not made it back to the manuscript in the methods section (so it appears it is not attended).

Answer: thanks for your insight and Code and meaning units were identified by three of the investigators (SH, HL and KC) and we set together and decide on the identified meaning units. And it is included in the revised version of the manuscript.

Line 215 – table – include information about how long midwives or if midwives have experience in a midwifery led model of care. This is important as later midwives discuss the success of this and would demonstrate expertise in delivery of this model of care.

Answer: since there is no well-established and recognized midwife-led continuity of care model in our country we just take the experience of midwives working in maternity unit. But, informally the midwife can give midwife-led continuity of care for example the midwife who provide the antenatal care might have the chance to attend the delivery of the mother and early postnatal care. But this would happen rarely because this would happen when the antenatal care midwife assigns in labour and delivery room during night time or weekend. Due to this reason considering the midwives experience in a midwife-led continuity of care would be difficult.

Line 217+ Can you demonstrate support for this overarching themes with participant quotes?
Answer: thank you so much for this and the following quote is used and included in the revised manuscript: *“As a professional midwife I would be happy if I can give all antenatal, labour and delivery and postnatal care for mothers using the continuum of care model. But, the reality is if I am assigned at antenatal care clinic I would only provide antenatal care; the other unit of care will be covered by other professionals” (midwife code 1 Mehal Meda Hospital).*

Terminology “non-midwife” do you mean doctor? Needs to be consistent thought the whole

manuscript.

Answer: With regard to “non-midwife” we used this terminology to represent other health care providers out of midwives who are working in maternal health care unit. It would include medical doctors, integrated emergency surgical officers, etc. and we include this in the revised manuscript at the sample size subsection

Q9 General comments not all of these have made it to the manuscript. Some are out of order eg authors responsible for data analysis should be in the methods section – it is included in list of author contributions.

Answer: we have included author’s contribution in the method section on the revised manuscript. We tried to include all the comments provided by the reviewers in the revised manuscript.

Thank you so much once again for your valuable comments and time you spent to evaluate our research work.

With kind regards,

Solomon Hailemeskel (PI)
