# Supplementary material for: Health care providers’ perceptions and experiences related to Midwife-led continuity of care–A qualitative study
Source: PLoS One. 2021 Oct 14;16(10):e0258248. doi: 10.1371/journal.pone.0258248 (PMC8516211; doi:10.1371/journal.pone.0258248)
Supplement: Attachment — Submitted filename: R2R.odt [file pone.0258248.s005.odt]

On Sat, Jul 3, 2021 at 7:04 AM solomon hailemeskel wrote:
Dear Dr. Louise Franklin,
Greetings!
First and foremost I would like to say thank you so much for your information. But all of the above comments given by the reviewer were responded to with the previous submission of revised manuscript (we the authors have submitted the second revised version of the manuscript with and without track change on June 13/ 2021. we have also prepared and submitted independent letters for response to reviewers).  What makes me confused is the same comment is given for us to make a minor revision. there is no new comment given from the reviewers to make revision. Hence, would you please give me your suggestion and direction to make a revision again.
Here I have attached the previous response given to the reviewer's comment and revised manuscript with track changes for your reference.
with kind regards,

------------------------------------------------------------------------------------------------------------------------------------------------------------------------------------------------------------------------------------------------------------------------------------

Solomon Hailemeskel (BSc. MSc)
PhD student at University of Gondar in Midwifery and Women's Health
Email: solomonhailemeskel9@gmail.com, solomonhmeskeldb@yahoo.com
Tel: +251(0)913312912
Debre Berhan University, Ethiopia
On Wed, Jun 30, 2021 at 10:27 PM Louise Franklin wrote:
PONE-D-20-34013R2
Health care providers? perceptions and experiences related to Midwife-led continuity of care - a qualitative study
PLOS ONE

Dear Mr Beshah,

Following your recent email, your Editor has replied witht he missing comments:


=====================

The manuscript is coming along and reads better this version.  A few minor ammendments are suggested to improve further for publication standard:
Line 91 & 92 needs reference
Line 98 when did ?traditional? obs/hosp care start
Line 147 ? for those who birth at home is it midwifery led?
Line 163  Details about research team: a) what are interviewers experience and training in interviewing for data collection?
b) Relationship with the participants?  Are any known to researcher? What relationships existed or were established around this work.

Line 172 ? overview broad question asked to the group to context for the reader
Line 185 ? Were participants given opportunity to provide feedback/ amend/  approve their transcripts prior data analysis?
Line 186 how many & who involved in coding? ? I note this was raised by another reviewer also.  Your response is provided in the response to reviewers but has not made it back to the manuscript in the methods section (so it appears it is not attended).
Line 215 ? table ? include information about how long midwives or if midwives have experience in a midwifery led model of care.  This is important as later midwives discuss the success of this and would demonstrate expertise in delivery of this model of care.
Line 217+ Can you demonstrate support for this overarching themes with participant quotes?
Terminology ?non-midwife?  do you mean doctor?  Needs to be consistent thought the whole manuscript.

Q9 General comments not all of these have made it to the manuscript. Some are out of order eg authors responsible for data analysis should be in the methods section ? it is included in list of author contributions.


=====================


I hope this is of assistance, we appreciate your patience
