# Supplementary material for: Health care providers’ perceptions and experiences related to Midwife-led continuity of care–A qualitative study
Source: PLoS One. 2021 Oct 14;16(10):e0258248. doi: 10.1371/journal.pone.0258248 (PMC8516211; doi:10.1371/journal.pone.0258248)
Supplement: Attachment — Submitted filename: Response to reviwers.docx [file pone.0258248.s006.docx]

**Answer to the editor's comments:**

A rebuttal letter, with point-by-point responses to yours and the reviewers' comments is provided in this revised version of the manuscript. The following order has been applied for the submission: Response to Reviewers; Revised Manuscript with Track Changes and Manuscript without track change.

**Journal Requirements:**

Please review your reference list to ensure that it is complete and correct. If you have cited papers that have been retracted, please include the rationale for doing so in the manuscript text, or remove these references and replace them with relevant current references. Any changes to the reference list should be mentioned in the rebuttal letter that accompanies your revised manuscript. If you need to cite a retracted article, indicate the article’s retracted status in the References list and also include a citation and full reference for the retraction notice.

Answer: yes we revised the reference list based on the journal reference style and we used endnote reference software. We have also tried to replace some of the old references with the recent and most appropriate one. We have checked all the references for retracted paper and all reference lists we used were not retracted. And we have actually corrected the reference lists based on pervious comments and we now check it again for any.

**Letter to reviewers**

**Review Comments followed by answers**

Reviewer #1: The manuscript continues to be refined and is much improved from earlier versions, so well done. However, there are still minor amendments which will improve the quality of your work and experience of the reader.

As per earlier feedback, language needs to be consistent, this occurs in many instances in your manuscript (as follows) so it needs to be accurate if they are midwives and doctors then continue with this terminology. If you include a tracked changed document these will be easily noted.

**Author’s response:** thank you so much for your valuable comments. For your general understanding we interview all health care providers working in maternal health care unit. In Ethiopian context the health care providers working in maternal health care unit are midwives, nurses, medical doctors and emergency surgical officers. For this reason we tried to classify the health care providers as midwives (who are professional midwives working in the maternal health care unit) and non-midwives (health care providers other than midwives who are currently working in maternal health care unit. This includes: medical doctors and emergency surgical officers). As you remember our research question was to answer the perception and experience of health care providers working in maternal health care unit about midwife-led continuity of care model. During our visit at the health facility we found 12-15 midwives in each hospital and 2 emergency surgical officers and 2 medical doctors working in maternal health care unit. As a result of this we conducted FGD for midwives and individual in-depth interview for emergency surgical officers and medical doctors. Finally, for write up and communication purpose we classify them as midwife and non-midwife. The main reason we use the term non-midwife was the health care providers out of midwives are not purely a medical doctors (some of them are medical doctors and some of them are emergency surgical officers). Hence, if this classification creates confusion for readers we better to keep the classification as midwife and integrated emergency surgical officers and medical doctors as it is.

LINE 30 “25 midwives and 8 emergency surgical officers and medical doctors”

Author’s response: Thanks for this and we keep is as it is

Versus

LINE 41 “25 midwives and the group of 8 non-midwives”

Author’s response: Thanks for this and we replaced the word non-midwives by emergency surgical officers and medical doctors

Versus

LINE 160 “25 midwives and 8 non-midwives (health care providers other than midwives working in maternal health care unit)”

Author’s response: Thanks for this and we replaced the word non-midwives by emergency surgical officers and medical doctors

versus
LINE 171 “ (non-midwife health care providers working in the maternal health care unit”

Author’s response: Thanks for this and we replaced the word non-midwives by emergency surgical officers and medical doctors

Versus

LINE 219 “25 midwives and 8 medical directors and integrated emergency surgical officers”

Author’s response: Thanks for this and we keep as it is

Versus

table 1 : “Medical Doctor Integrated Emergency Surgical Officer (IESO)”

Author’s response: Thanks for this and we keep as it is

LINE 299 Non-midwife

Author’s response: Thanks for this and we replaced the word non-midwives by emergency surgical officers

LINE 325 Non-midwife

Author’s response: Thanks for this and we replaced the word non-midwives by medical doctors

LINE 3xx Non-midwife

Author’s response: Thanks for this and we replaced the word non-midwives by emergency surgical officers

LINE 352 Non-midwife

Author’s response: Thanks for this and we replaced the word non-midwives by emergency surgical officers and medical doctors

LINE 357 non-midwife health care providers

Author’s response: Thanks for this and we replaced the word non-midwives by emergency surgical officers and medical doctors

LINE 369 non-midwife health care provider.

Author’s response: Thanks for this and we replaced the word non-midwives by emergency surgical officers and medical doctors

LINE 374 non-midwife

Author’s response: Thanks for this and we replaced the word non-midwives by medical doctors

LINE 405 non-midwife = medical model

Author’s response: thanks for the comments and we replaced the word non-midwife-led model by existed model of care (shared model of care).

Conclusion:

LINE 30 seems most accurate

LINE 87 do you have a reference for your improved care outcomes?

Author’s response: thanks for the comment and appropriate reference is inserted in the document

LINE 124 So this study explores midwives and medical doctors perceptions. Be clear this is who is included…. Unless there are other health care professionals included? Then specify these.

Author’s response: thanks for your comments. Just to be clear we tried to include all health care providers who are working in maternal health care unit. For this reason we interviewed medical doctors and integrated emergency surgical officers. And we do FGD with midwives. The main reason we cannot categorize the participants as midwives and medical doctors were the integrated emergency surgical officers are not a medical doctor. They specialize from other health science professional like nurses and health officers at master’s level to do emergency surgical procedures like emergency caesarian section and acute abdomen. Due to this reason we choose the word non-midwife for them. However we replaced the pervious word by integrated emergency surgical officers and medical doctors. And correction made accordingly

LINE 162. Needs re-writing for clarity “At this saturation point, the study participants were unable to provide any additional new ideas”.

Author’s response: thanks for the comment and it is rewrite like this “The data collection was completed when no new/unique information was emerged”

LINE 179 edit for clarity

Author’s response: The discussion was initiated by asking the study participants to explain about the type of model of maternal health care practiced in their hospitals and how do they understand model of maternal health care? This sentence was the first open ended question asked for study participants to initiate the discussion. Then following their response we ask them a probing question to explore more.

LINE 188 Not clear if returned to participants for checking?

Author’s response: All the interviews were performed and coded initially by the first author, who was fluent speaker in original language (Amharic). Hence, we did not return the translated word to the participants.

LINE 223 Comment if this is the usual distribution of genders for midwives (ie are they mostly male???)

Author’s response: thank you so much for this and it seems stranger. But, according to state of Ethiopian midwives study the distribution of female midwives was 67.3%. However, we found small number of female midwives in our study area during the time of interview.

LINE 493 “This study is the first to describe the experience and perceptions of” use which ever term you choose consistently and conclude with it here eg Midwives and doctors

Author’s response: thanks for the comment. We used the term Ethiopian health care providers because we include all types of health care providers working in maternal health care unit. But to be more clear on the conclusion we change the word and replaced by “Midwives, Integrated emergency surgical officers and medical doctors”

Thank you so much once again for your valuable comments and time you spent to evaluate our research work.

With kind regards,

Solomon Hailemeskel (PI)
